# School Collective Efficacy and Bullying Behaviour: A Multilevel Study

**DOI:** 10.3390/ijerph14121607

**Published:** 2017-12-20

**Authors:** Gabriella Olsson, Sara Brolin Låftman, Bitte Modin

**Affiliations:** Centre for Health Equity Studies, Stockholm University/Karolinska Institutet, Gabriella Olsson 10691, Sweden; sara.brolin.laftman@chess.su.se (S.B.L.); bitte.modin@chess.su.se (B.M.)

**Keywords:** victimization, perpetration, peer aggression, collective efficacy, contextual, school

## Abstract

As with other forms of violent behaviour, bullying is the result of multiple influences acting on different societal levels. Yet the majority of studies on bullying focus primarily on the characteristics of individual bullies and bullied. Fewer studies have explored how the characteristics of central contexts in young people’s lives are related to bullying behaviour over and above the influence of individual-level characteristics. This study explores how teacher-rated school collective efficacy is related to student-reported bullying behaviour (traditional and cyberbullying victimization and perpetration). A central focus is to explore if school collective efficacy is related similarly to both traditional bullying and cyberbullying. Analyses are based on combined information from two independent data collections conducted in 2016 among 11th grade students (*n* = 6067) and teachers (*n* = 1251) in 58 upper secondary schools in Stockholm. The statistical method used is multilevel modelling, estimating two-level binary logistic regression models. The results demonstrate statistically significant between-school differences in all outcomes, except traditional bullying perpetration. Strong school collective efficacy is related to less traditional bullying perpetration and less cyberbullying victimization and perpetration, indicating that collective norm regulation and school social cohesion may contribute to reducing the occurrence of bullying.

## 1. Introduction

In a school context, bullying is one of the most common expressions of violence [1]. Being exposed to bullying is often a pervasive experience that impacts both short- and long-term social, behavioural, and psychological development [2,3,4,5]. Bullying behaviour has, for instance, commonly been reported as an important risk factor for suicide in adolescence, both directly [6,7] and indirectly, by its influence on other risk factors linked to suicidal thoughts and behaviours [8,9,10]. In addition, studies suggest that the problem of bullying extends beyond the victim-perpetrator(s) relationship since its occurrence in a school class appears to involve negative health consequences also for those who are not directly involved [4,11,12]. Bullying is often defined as repeated negative actions by peers and presumes a power imbalance between the perpetrator(s) and the victim [13]. These negative actions may be social, physical or verbal, direct or indirect, but serve the common purpose of intentionally injuring or causing the victim discomfort. Bullying takes place not only in “face-to-face” interactions but also via mobile phones and the Internet. In the present paper, the former type of bullying is referred to as traditional bullying, while the latter is defined as cyberbullying. Cyberbullying is a relatively new form of bullying. Although the definition of cyberbullying often relies on Olweus’s [13] criteria, it differs from traditional bullying in a number of ways. A perpetrator can, for instance, be anonymous and the impact and spread of an incident may be more pervasive when resulting from cyberbullying rather than traditional bullying [14]. Earlier research suggests that students´ involvement in cyberbullying and traditional bullying overlaps to some extent [6,15,16,17]. Nevertheless, several studies have demonstrated significant associations between cyberbullying and psychological health outcomes, also when adjusting for traditional forms of bullying. This might suggest that cyberbullying is distinct from other types of bullying, and that its effects on health also may be different. Cyberbullying is generally less studied than other forms of bullying and, in addition, few studies have compared effects across outcomes while making a clear distinction between bullies and victims [14]. Given the complex nature of bullying and the fact that cyberbullying only partly overlaps traditional bullying behaviours, it is important to explore them separately [14,15].

As with other forms of violent behaviours, bullying is the result of multiple influences acting at different societal levels [18]. Yet the majority of studies on bullying focus primarily on the characteristics of the perpetrators and the victims [11,19]. Although interest in contextual effects has increased in recent years, relatively few studies have explored how characteristics of central contexts in young people’s lives are linked to bullying behaviours on the individual level. Yet there is evidence suggesting that factors on multiple levels need to be considered for a fuller understanding of why such behaviours arise [11,18,20]. Given that several intervention programs have been designed to reduce bullying by modifying the school environment, the lack of school contextual research is particularly problematic [21,22].

### 1.1. School Effects on Bullying Behaviour

Schools are well-defined units characterized by enduring social relationships. As such, schools are ideal for exploring how the social interplay between young people is affected by the larger social context in which they are embedded [23]. Regardless of whether bullying occurs electronically or “face-to-face”, it often stems from impaired relationships in schools. As such, cyberbullying is not simply a consequence of new technologies but, rather, a new dimension of a problem that has existed for a long time [14,23]. Most of the studies that have explored school effects on bullying behaviours have looked at how the structural characteristics of schools are related to bullying experiences [11,18,22]. For instance, in these types of studies, school-level indicators of disorder, such as student-teacher ratio, concentration of student poverty, student mobility, and suspension rates, have been found to be significant predictors of bullying-related attitudes and experiences [20,24]. Far fewer studies have investigated the social processes linking aspects of school structure to bullying behaviours [22]. Nonetheless, a recent review [24] supports the idea that it is also important to take into consideration a school’s social environment in relation to bullying behaviours. Studies concerning the influence of schools’ social environments and social processes in relation to bullying behaviours have often explored different aspects of the school climate [23,25], that is, the collective beliefs, values, and attitudes that permeate a school and which are produced and reproduced in the social relationships between students, teachers, and other school staff [26]. However, the concept of school climate has been criticized as being vague and imprecise and as it is often defined differently across studies, findings are inconclusive [23,24]. A related and clear limitation of school contextual studies, not only generally [27], but also specifically regarding bullying behaviours, is that they are seldom theoretically driven [23]. Hence, they provide little rationale for understanding the school effects revealed. Therefore, research using theoretically derived concepts that can help to specify the process by which school contexts influence student bullying has been called for [23].

The theoretically derived concept of collective efficacy offers a lens through which certain, more precise, components of a school’s social environment can be examined in relation to bullying behaviours. Building on ideas derived from neighbourhood studies of structural and demographic determinants of crime [28] and theories of social capital [29], collective efficacy was originally conceptualized as a neighbourhood property that defines the willingness of local residents to intervene for the common good, for example, in questions of public order or controlling crime [30,31,32]. Variation in the ability to exert collective action across neighbourhoods is assumed to be an important mechanism in the association between neighbourhood structural characteristics and crime rates [28,30]. Building on these ideas, Sampson [32] introduced the concept of collective efficacy to the field of neighbourhood studies and crime as an attempt to empirically explore the hypothesized social process that links structural disadvantage to crime rates [29,31]. The concept rests on two central components: social cohesion (i.e., levels of trust between residents) and informal social control (i.e., neighbourhood residents’ conjoint ability and readiness to intervene for the common good). According to the theory, the community members’ conjoint willingness to intervene is thought to largely depend on conditions of mutual trust. Thus, socially cohesive neighbourhoods are seen as the most fertile contexts for the realization of informal social control [31]. In line with these theoretical ideas, neighbourhood collective efficacy has been found to be negatively associated with several measures of violence, even after controlling for individual-level characteristics, and to mediate the effects of neighbourhood disadvantage on criminal outcomes [31,32]. Although originally applied to neighbourhoods and crime, the logic of collective efficacy has also been fruitfully extended to other contexts and outcomes, including bullying and school settings [19,22,33]. Applied to the school setting, collective efficacy refers to the degree of social cohesion and informal control in the school. Hence, rather than referring to some kind of formal or disciplinary control, schools with a strong collective efficacy is characterized by mutual trust and supportive relationships as well as a shared willingness to take on a responsibility of protecting or promoting the common good [22]. Studies by Sapouna [34] and Williams and Guerra [23] provide some initial evidence that the concept of collective efficacy can also contribute to the understanding of exposure to and victimization of bullying in the school context. Sapouna [34] found that individual-level victimization (but not perpetration) was more frequent in school classes with lower levels of collective efficacy, even after controlling for individual characteristics; Williams and Guerra [23], on the other hand, found bullying perpetration to be less likely in schools characterized by cohesion and trust. Bullying perpetration was also found to be less likely in schools characterized by the perception that others, particularly teachers, would intervene to prevent bullying.

### 1.2. Aim of the Study

The aim of the current study is to assess whether teacher-reported school collective efficacy, referring to the degree of social cohesion and informal social control within a school, is related to student-reported exposure to or perpetration of bullying. A central focus is the exploration of whether school collective efficacy is related similarly to both traditional bullying and cyberbullying. To this end, we combine newly collected data from two separate sources: the Stockholm Teacher Survey and the Stockholm School Survey. It is hypothesized that strong collective efficacy at the school level is associated with less bullying victimization and perpetration at the student level, with regard to both traditional bullying and cyberbullying. 

## 2. Materials and Methods

The study is based on data that combines information from students and teachers in upper secondary schools (all state schools and the majority of independent schools) in Stockholm municipality. Student level information is based on responses from 11th grade students (aged 17–18) who took part in the 2016 Stockholm School Survey (SSS). The SSS is conducted biennially in Stockholm municipality. Participation is mandatory for public schools; independent schools take part on a voluntary basis. The survey is distributed in classrooms by teachers and the completed questionnaires are returned in sealed envelopes. In addition, school contextual information from the Stockholm Teacher Survey (STS) of 2016 was linked to the SSS data. The STS was conducted among all teachers working in upper secondary schools in Stockholm municipality (*n* = 2501) with the primary purpose of gathering information about schools’ learning, working, and social environments. The pooled data used in the present study consists of information from students and teachers in 21 public and 37 independent schools. In all, the data set comprises information from 6067 responding students (public = 3544 and independent = 2523), and 1251 responding teachers (public = 640 and independent = 611). This corresponds to a response rate of 69% for schools, 72% for students, and 51% for teachers. External attrition among schools and teachers is due to schools not participating in at least one of the surveys; mostly this refers to smaller independent schools with few students but also two public schools. Student attrition is due to absence from school the day of the survey and internal non-response due to unreliably filled-in questionnaires. Due to high non-response rates in relation to some of the outcome measures (traditional bullying perpetration *n* = 345 (5.69%), cyberbullying victimization *n* = 410 (6.76%), and cyberbullying perpetration *n* = 286 (4.71%)), four sets of outcome-specific analytical subsamples were created. The final analytical subsamples consist of 58 schools with *n* = 6067 for traditional bullying victimization, *n* = 5722 for traditional bullying perpetration, *n* = 5657 for cyberbullying victimization, and *n* = 5781 for cyberbullying perpetration. 

The Regional Ethical Review Board of Stockholm gave permission for the STS (2015/1827-31/5). Since the SSS is completed anonymously and no personal identification information is provided, the Regional Ethical Review Board of Stockholm is not considering the data for ethical approval (2010/241-31/5).

### 2.1. Variables

#### 2.1.1. Dependent Variables

Bullying and cyberbullying behaviours were measured by questions from the Stockholm School Survey. Traditional bullying victimization was measured by the question: “How often have you been bullied or harassed at school this year?” Students who responded “I haven’t been bullied” or “It’s happened occasionally” were classified as not having been subjected to traditional bullying victimization, whereas those who responded “2 or 3 times a month”, “About once a week”, or “Several times a week”, were classified as having been subjected to traditional bullying victimization. Since the purpose of the study was to capture bullying among peers, students who, in a previous question, had marked that they were bullied by teachers, but not by peers, were classified as not subjected to traditional bullying victimization.

Traditional bullying perpetration was measured by the question: “Have you taken part in the bullying or harassment of other students this school year?” Students who responded “No” or “Yes, occasionally” were classified as not being perpetrators of traditional bullying, whereas those who responded “Yes, 2 or 3 times a month”, “Yes, about once a week”, or “Yes, several times a week” were classified as being involved in traditional bullying perpetration. Those who responded “Don’t know” were classified as no-response.

Cyberbullying victimization was measured by the question: “Have you been bullied or harassed by someone on the Internet or by text message (SMS/MMS) this school year?” The response categories were “Yes”, “No”, and “Don’t know”; responses in the last category were classified as no-response.

Cyberbullying perpetration was measured by the question: “Have you taken part in the bullying or harassment of other students on the Internet or by text message this school year?” The response categories were “Yes”, “No”, and “Don’t know”, and responses in the last category were classified as no-response. The two questions on cyberbullying were asked immediately after a set of questions on traditional bullying behaviour, in which examples of various forms of traditional bullying victimization were clearly specified (e.g., if the student had been teased, been frozen out, physically hurt, threatened or forced to do things). Thus, the respondents were probed with practical examples of the meaning of traditional bullying that also facilitates the distinction between traditional bullying and cyberbullying.

#### 2.1.2. Independent Variables (School-Level)

School collective efficacy was constructed from an index based on the responses to the following four statements from the STS: “At this school, adults would intervene, even outside the classroom, if the school rules were being broken”; “At this school, graffiti and vandalism are unusual”; “This is a close-knit school”; and “People at this school can be trusted”. The response categories were on a five-point scale: “Strongly agree” (5); “Agree” (4); “Neither agree nor disagree” (3); “Disagree” (2), and; “Strongly disagree” (1). The responses to the four statements were added to a scale ranging from 4 to 20, with higher values indicating stronger collective efficacy. Exploratory and confirmatory factor analyses demonstrate good model fit (Root mean squared error of approximation (RMSEA) = 0.052, Tucker-Lewis index (TLI) = 0.995, Comparative fit index (CFI) = 0.998), and the internal consistency of the items was sufficient (Cronbach’s alpha = 0.74). The mean value of teachers’ responses in each school was used to measure school collective efficacy at the school level, and linked to the student-level data. In order to detect any potential non-linear associations between school collective efficacy and bullying behaviour, these school mean values of collective efficacy were divided into three groups of approximately equal size, to distinguish between schools with strong (32.76% of all schools), intermediate (32.76% of all schools), and weak collective efficacy (34.48% of all schools).

#### 2.1.3. Control Variables (Individual-Level)

Gender was measured by the question: “Are you a boy or a girl?” and the response categories “Boy” and “Girl”. Owing to high frequencies of bullying victimization among students who did not respond to this question (3.85%), the responses were placed in a separate “Not reported” category.

Family structure was measured by the question “Who do you live with?” with a list of options to choose from. Those who responded both “Mother” and “Father” were classified as living with two parents in one household and were compared with all others.

Parents’ university education was measured by the question: “What is the highest level of education your parents have?” The response categories, which covered mother and father individually, were: “Old elementary school or compulsory school (max 9 years schooling)”; “Upper secondary school”; “University and/or university college”, and; “Don’t know”. Those who responded “University and/or university college” for at least one parent were classified as having one parent with a university education and were compared with all others.

Migration background was measured by the question: “How long have you lived in Sweden?” The response categories were: “All my life”; “10 years or more”; “5–9 years”, and; “Less than 5 years”. Those who responded that they had lived in Sweden less than nine years were grouped (due to small numbers) and compared with the two other categories.

### 2.2. Statistical Method

Since the data were hierarchical with students nested in schools, multilevel modelling was applied. Multilevel models takes the hierarchical structure of the data into account by allowing the variance in the outcomes to be separated between higher and lower level units, in this case into student-level variation and school-level variation. The proportion of the total variance contributed by the school-level variance component were assessed by means of ‘‘rho’’ estimates (similar to the intraclass correlation). Two-level binary logistic regression models were estimated using Stata 14 and the *xtlogit* command. Odds ratios (ORs) with 95% confidence intervals (CIs) are reported. The OR refers to “the odds that an outcome will occur given a particular exposure, compared to the odds of the outcome occurring in the absence of that exposure” [35] (p. 27).

## 3. Results

Descriptive statistics are presented in Table 1. In the sample, about 2% of the students reported that they had been exposed to traditional bullying; 1% reported that they had engaged in traditional bullying perpetration; 7% reported cyberbullying victimization; and 3% reported cyberbullying perpetration. School-level statistics further suggest that of the included students, 25% attended a school characterized by strong teacher-rated collective efficacy, 36% attended a school characterized by intermediate collective efficacy, and 38% of the students a school with weak teacher-rated collective efficacy.

School-level statistics presented in Table 2 suggest that the proportion of students reporting victimization of or perpetration of bullying behaviours varies across schools. The variation is most substantial in relation to the measures of victimization. The proportion of students who reported that they had been subjected to traditional bullying ranged from 0 to 20% across schools. In relation to cyberbullying victimization, the proportion ranged from 0 to 25% across schools. The proportion of students reporting traditional bullying perpetration varied from 0 to 6.7% across schools, and for cyberbullying perpetration, from 0 to 11.7%.

Table 3 presents mean values and ranges for school collective efficacy, and the proportions of students who reported being victims or perpetrators of either traditional bullying or cyberbullying, by the schools’ level of collective efficacy. For all measures of bullying behaviour, although somewhat less clear in relation to traditional bullying victimization, there are gradients in the expected directions. Thus, the stronger the level of collective efficacy in the school, the smaller the proportion of students reporting traditional bullying perpetration or cyberbullying victimization and perpetration. Traditional bullying victimization is most frequently reported by students in schools with weak collective efficacy, and least frequently reported by students in schools with an intermediate level of collective efficacy. Chi-square tests show that the pattern is statistically significant in relation to all bullying outcomes, but it is most clear in relation to cyberbullying.

Results from the multilevel analyses of traditional bullying behaviour, distinguishing the effects of individual-level characteristics from school-level characteristics, are presented in Table 4. First, an empty model is applied. As indicated by the intra-class correlation coefficient (rho), 7.6% of the total variation in traditional bullying victimization, and 5.3% of the total variance in traditional bullying perpetration can be attributed to differences between schools. Next, in Model 1, school-level collective efficacy is introduced. The inclusion of school collective efficacy reduces the school-level variance to 6.7% for traditional bullying victimisation and to 2.9% for traditional bullying perpetration, thus suggesting that part of the school variation in bullying victimization and, in particular, bullying perpetration can be attributed to school collective efficacy.

Furthermore, the results of Models 1 demonstrate that students in schools with intermediate collective efficacy are less likely to report traditional bullying victimization than those in schools with weak collective efficacy (OR = 0.58, 95% CI 0.33**–**0.99). There is, however, no statistically significant difference in the likelihood of reporting traditional bullying victimization between students attending schools with weak vs. strong collective efficacy (OR = 0.77, 95% CI 0.44**–**1.37). With regards to traditional bullying perpetration, the result suggests that students in schools with strong collective efficacy are less likely to report such behaviours compared to students in schools with weak collective efficacy (OR 0.29, 95% CI 0.11**–**0.74). In Models 2, student-level variables are added. The results demonstrate that the estimates of school collective efficacy remain largely similar in size, suggesting that school collective efficacy is associated in particular with bullying perpetration, even when adjusting for individual-level characteristics.

Results from the multilevel analyses on cyberbullying are presented in Table 5. The empty models suggest that a statistically significant part of the total variation in cyberbullying victimization and cyberbullying perpetration, respectively, can be attributed to differences between schools: 3.0% for cyberbullying victimization, and 6.2% for cyberbullying perpetration. In Models 1, including school collective efficacy reduces the school-level variance to 2.2% for cyberbullying victimization and to 0.9% for cyberbullying perpetration. This suggests that school collective efficacy contributes substantially to explaining school-level differences, and particularly so for cyberbullying perpetration. Models 1 further suggest that schools’ levels of collective efficacy are negatively associated with both cyberbullying victimization and perpetration. Specifically, the results suggest that students in schools characterized by strong levels of collective efficacy are less likely to report that they are victims (OR 0.65, 95% CI 0.46**–**0.92) or perpetrators (OR 0.32, 95% CI 0.19**–**0.54) of cyberbullying compared to students in schools characterized by weak levels of collective efficacy. These associations largely persist when student-level variables are added in Models 2.

## 4. Discussion

The current study sought to explore if teacher-rated school collective efficacy was related to student-reported bullying victimization and perpetration. The results indicated that strong school collective efficacy was related to less traditional bullying perpetration and less cyberbullying victimization and perpetration, confirming our hypothesis. For traditional bullying victimization, the results were less clear-cut since intermediate, but not strong, school collective efficacy demonstrated a tendency to be associated with less victimization.

The results correspond well with theory [28,32] and with the few previous studies on the topic [23,34]. By showing that strong collective efficacy is clearly and independently associated with lower levels of bullying, this study gives further support to the idea that school environments that are perceived as safe and characterized by collective regulation of behaviours contribute to reducing the occurrence of problem behaviours [36,37] and, more specifically, bullying behaviours [23,34]. Moreover, in line with results from other studies [23,36,37], the findings suggest that the concept of collective efficacy is not only applicable, as originally theorized [28,32], in neighbourhood settings but can also be extended to other contexts. In fact, it has been suggested previously [23] that the collective efficacy concept may be particularly useful for understanding contextual influences on social behaviours when contexts, such as in schools, are defined and when monitoring and regulating behaviour is an important function of individuals in that context.

A key focus of this study has been to explore if school collective efficacy is similarly related to both traditional bullying and cyberbullying. Although the number of studies exploring cyberbullying is growing fast [38,39], the concept of school collective efficacy has not, to our knowledge, been explored in relation to cyberbullying before. Thus, by showing that school collective efficacy contributes clearly to the occurrence of cyberbullying victimization and perpetration, this study adds to existing knowledge. Furthermore, the findings suggest that cyberbullying is not only more prevalent than traditional bullying among 11th graders in schools in Stockholm, but that school collective efficacy is also more clearly related to cyberbullying than to traditional bullying. Moreover, in line with what has been suggested elsewhere [14] the strong association between school collective efficacy and cyberbullying points to the idea that cyberbullying does, first and foremost, stem from impaired relationships and school circumstances and that it is not primarily a consequence of increased access to new media. As indicated by the results of the current study, school interventions aimed at strengthening levels of collective efficacy may thus be one way to reduce the occurrence of cyberbullying.

In all, the results suggest that the willingness of people to intervene in instances of bullying may be facilitated in schools characterized by high levels of trust and social cohesion. Nonetheless, it is central to keep in mind that the concept of collective efficacy rests on the idea of shared norms. Accordingly, from a school preventive point of view, it is important to ensure that schools are also characterized by sound norms against bullying. Otherwise, as suggested by research on social capital [40], strong ties could encourage adverse attitudes and behaviours. In contrast to what might be intuitively expected, it is not self-evident that schools are always characterized by strong anti-bullying norms. On the contrary, research suggests that norms regarding bullying may vary across schools [1]. In schools characterized by more pro-bullying types of attitudes, intervening in instances of bullying may not be perceived as the way to act “for the common good”, but may instead be associated with adverse social outcomes [1]. Previous research suggests that changing the attitudes and behaviours of teachers may be one way to foster sound norms and set the standard for social relationships for schools as a whole [11,41]. Students’ perceptions that teachers clearly disapprove of bullying and intervene in incidents of bullying has for instance repeatedly been linked to less bullying in schools [11,41]. Moreover, as suggested by the collective efficacy literature the willingness to intervene for the common good is related to levels of trust in the setting in question [32]. As such, strengthening levels of collaboration between teachers [42] and striving towards supportive relations between teachers and between teachers and students [25,43] may further enhance the motivation to intervene and report incidents of bullying in school settings. In addition, it has been suggested that teachers’ beliefs about the causes of bullying also influence whether they intervene in relation to incidents of bullying or whether bullying factors are attributed to be outside of their control [11]. Hence, strengthening teachers’ awareness of bullying as a contextual problem that not only relates to the involved individuals and their characteristics may thus further enhance their motivation to act against bullying.

### Strengths and Limitations

This study was based on new and unique data material covering a substantial portion of 11th grade students and schools in Stockholm, making it ideal for multilevel analyses. Furthermore, the fact that the study uses two separate data sources with different informants limits the risk of shared methods variance. However, the fact that data were collected in Stockholm makes generalization to the general Swedish population or other populations less straightforward. Moreover, high internal non-response to questions on bullying, as well as external non-response due to student absence on the days of the surveys, may have biased the analyses. It might, for instance, be reasonable to believe that (a) those directly involved in bullying are less willing to report this and that (b) absent students are more involved in problem behaviours than others. In other words, neither external nor internal non-response can be claimed to be completely at random. There may also be potential confounders. As discussed previously, school norms in relation to bullying may be such a confounder; gender may be another. Simply put, boys are often found to be more involved in bullying than girls. However, findings are inconsistent and seem to vary depending on the type of bullying, physical, verbal, traditional, or cyberbullying, being explored [6,11,12]. The effect of collective efficacy has also been shown to be gender-specific in the sense that boys tend to be less likely to engage in bullying perpetration when classroom collective efficacy is rated as strong [34]. In relation to both the potential confounders mentioned above, further research would be enlightening. Finally, longitudinal research exploring the relationships studied in this paper is desirable. The fact that this study is based on cross-sectional data makes it impossible to claim that collective efficacy leads to less bullying. The opposite could, empirically at least, just as well be true.

## 5. Conclusions

In conclusion, the risk of being a perpetrator of traditional bullying or a victim or a perpetrator of cyberbullying appears to be lower in schools characterized by strong collective efficacy, and higher in schools characterized by weak collective efficacy, regardless of students’ sociodemographic background characteristics. As a result, in terms of recommendations for school practice, it is essential to strengthen awareness among students and school staff that bullying is not a relational problem relevant only to those immediately involved; it is a problem that concerns the school as a whole. Consequently, school-based interventions should be designed to address the broader school environment and not only individual-level processes. In line with what is suggested in the collective efficacy literature, striving towards a school environment characterized by norms that clearly disapprove of bullying and in which it is self-evident to intervene, inside and outside the classroom, if somebody is being bullied may be one way forward.

## Figures and Tables

**Table 1:** Descriptive statistics of variables included in the analyses. *n* = 6067 students in 58 schools.

Independent Variables	*n*	%	*n*	%
*Individual-level*				
Bullying				
Traditional bullying victimization			139	2.30
Traditional bullying perpetration ^a^			62	1.08
Cyberbullying victimization ^b^			411	7.27
Cyberbullying perpetration ^c^			174	3.01
Gender				
Boys			2798	46.12
Girls			3064	50.50
Not reported			205	3.38
Family structure				
Two parents in the same household			3777	62.25
Other			2290	37.75
Parents university educated				
No parent			2068	34.09
At least one parent			3999	65.91
Migration background				
Lived in Sweden all life			4937	81.37
Lived in Sweden ≥10 years			530	8.74
Lived in Sweden <10 years			600	9.89
*School-level*				
School collective efficacy	*n* ^student^	% ^student^	*n* ^school^	% ^school^
Weak	2341	38.59	20	34.48
Intermediate	2205	36.34	19	32.76
Strong	1521	25.07	19	32.76

**^a^**: *n* = 5722; **^b^:** *n* = 5657; **^c^:** *n* = 5781.

**Table 2:** Proportions of students reporting bullying behaviours across schools (*n* = 58).

Bullying Behaviours	Mean (%)	Range (%)
Traditional bullying victimization	3.1	0–20.0
Traditional bullying perpetration	1.2	0–6.7
Cyberbullying victimization	8.0	0–25.0
Cyberbullying perpetration	2.9	0–11.7

**Table 3:** Mean values and ranges of school collective efficacy, and proportions of students reporting bullying behaviours, by thirds of school collective efficacy. *n* = 5722–6067 in 58 schools.

Categories of School Collective Efficacy	School Collective Efficacy		Traditional Bullying Victimization	Traditional Bullying Perpetration	Cyberbullying Victimization	Cyberbullying Perpetration
	Mean	Range	%	%	%	%
School collective efficacy	16.1	11.15–20.00				
Weak (ref.)	14.3	11.15–15.16	2.86	1.33	8.60	4.05
Intermediate	16.9	15.18–16.90	1.75	1.29	6.99	3.08
Strong	18.3	16.93–20.00	2.24	0.41	5.67	1.36
			*p* = 0.037	*p* = 0.017	*p* = 0.003	*p* = 0.000

**Table 4:** Traditional bullying victimization and perpetration regressed on school collective efficacy. Results from two-level binary logistic random intercept models. Odds ratios and 95% (confidence interval (CI)) reported.

Categories of School Collective Efficacy	Traditional Bullying Victimization (*n* = 6067)			Traditional Bullying Perpetration (*n* = 5722)		
School collective efficacy	Empty Model ^a^	Model 1 ^b^	Model 2 ^c^	Empty Model ^a^	Model 1 ^b^	Model 2 ^c^
Weak (ref.)		1.00	1.00		1.00	1.00
Intermediate		**0.58 (0.33–0.99)**	0.61 (0.36–1.02)		0.94 (0.52–1.71)	0.98 (0.54–1.81)
Strong		0.77 (0.44–1.37)	0.81 (0.47–1.39)		**0.29 (0.11–0.74)**	**0.37 (0.14–0.98)**
sigma_u (SE)	0.51886 (0.13)	0.48618 (0.13)	0.41799 (0.14)	0.42990 (0.25)	0.31330 (0.27)	0.22815 (0.38)
rho	**0.076**	**0.067**	**0.050**	0.053	0.029	0.016
−2 log-likelihood	−657.23	−655.29	−651.16	−341.64	−337.00	−319.35

**^a^** Empty model contains no independent variables; **^b^** Model 1 adds school collective efficacy; **^c^** Model 2 adds gender, family structure, parents’ university education, and students’ migration background. Estimates in bold are significant.

**Table 5:** Cyberbullying victimization and perpetration regressed on school collective efficacy. Results from two-level binary logistic random intercept models. Odds ratios and 95% (CI) reported.

Categories of School Collective Efficacy	Cyberbullying Victimization (*n* = 5657)			Cyberbullying Perpetration (*n* = 5781)		
School collective efficacy	Empty Model ^a^	Model 1 ^b^	Model 2 ^c^	Empty Model ^a^	Model 1 ^b^	Model 2 ^c^
Weak (ref.)		1.00	1.00		1.00	1.00
Intermediate		0.78 (0.58–1.06)	0.80 (0.59–1.07)		0.75 (0.53–1.07)	0.78 (0.56–1.09)
Strong		**0.65 (0.46–0.92)**	**0.67 (0.47–0.93)**		**0.32 (0.19–0.54)**	**0.39 (0.24–0.64)**
sigma_u (SE)	0.31957 (0.08)	0.27168 (0.08)	0.24444 (0.08)	0.46510 (0.13)	0.17338 (0.23)	0.00603 (0.08)
rho	**0.030**	**0.022**	**0.018**	**0.062**	0.009	0.000
−2 log-likelihood	−1467.05	−1464.08	−1453.46	−777.24	−768.56	−751.20

**^a^** Empty model contains no independent variables; **^b^** Model 1 adds school collective efficacy; **^c^** Model 2 adds gender, family structure, parents’ university education, and students’ migration background. Estimates in bold are significant.
